# Hierarchical Pooling in Graph Neural Networks to Enhance Classification Performance in Large Datasets

**DOI:** 10.3390/s21186070

**Published:** 2021-09-10

**Authors:** Hai Van Pham, Dat Hoang Thanh, Philip Moore

**Affiliations:** 1 School of Information and Communication Technology, Hanoi University of Science and Technology, 1 Dai Co Viet, Le Dai Hanh, Hai Ba Trung, Hanoi City 10000, Vietnam; thanhdath97@gmail.com; 2 School of Information Science and Engineering, Lanzhou University, Feiyun Building, 222 Tianshui S Rd, Chengguan Qu, Lanzhou 730030, China; ptmbcu@gmail.com

**Keywords:** knowledge graphs, graph classification, graph neural networks, graph convolutional network, hierarchical graph pooling, FPool

## Abstract

Deep learning methods predicated on convolutional neural networks and graph neural networks have enabled significant improvement in node classification and prediction when applied to graph representation with learning node embedding to effectively represent the hierarchical properties of graphs. An interesting approach (DiffPool) utilises a differentiable graph pooling technique which learns ‘differentiable soft cluster assignment’ for nodes at each layer of a deep graph neural network with nodes mapped on sets of clusters. However, effective control of the learning process is difficult given the inherent complexity in an ‘end-to-end’ model with the potential for a large number parameters (including the potential for redundant parameters). In this paper, we propose an approach termed FPool, which is a development of the basic method adopted in DiffPool (where pooling is applied directly to node representations). Techniques designed to enhance data classification have been created and evaluated using a number of popular and publicly available sensor datasets. Experimental results for FPool demonstrate improved classification and prediction performance when compared to alternative methods considered. Moreover, FPool shows a significant reduction in the training time over the basic DiffPool framework.

## 1. Introduction

A field of research which has gained significant traction is the application of Deep Learning (DL) inspired by Convolutional Neural Networks (CNN) [1]. DL has been shown to be a very powerful method because of its ability to handle large datasets. DL algorithms have demonstrated the capability to extract high-level complex abstractions as data representations through a hierarchical learning process [2]. Moreover, DL has been shown to be an effective method capable of solving many machine learning problems in diverse fields which include: image processing, natural language processing, and the video gaming industry. However, the data generated can result in large datasets represented in spaces with a finite number of dimensions in both two-dimensional (flat) and three-dimensional spaces.

While research has resulted in a large volume of published studies where many different graph convolutional layers for Graph Convolutional Networks (GCN) have been proposed, the number of proposed pooling layers remains small [1]. Notwithstanding this limitation, intelligent pooling of graphs is a promising direction for research given that (a) it can identify both feature-based and structure-based clusters, and (b) reduce the computational overhead required by reducing the number of nodes [1]. Taken together, these potential benefits “promise to abstract from nodes to sets of nodes” and are “also a stepping stone towards enabling Graph Neural Networks (GNN) to modify graph structures instead of only node features” [1].

GNN implementations are DL techniques applied to graphs and are effective for node representation in a broad range of fields [3]. Data sets with unstructured data types (including sensor data represented as graphs) have gained traction driven by the modelling capabilities of knowledge graphs (KG) in heterogeneous domains. Traditional graph classification methods are based on GNN; however, such methods generally fail to learn the hierarchical representation of graphs [4,5]. Two-dimensional graphs are inherently flat and only propagate information across edges of graphs resulting in a failure to capture hierarchical information. Lan et al. in [6] propose a novel complex fuzzy inference system using a KG with extensions designed to provide decision support. Viet et al. in [7] have introduced extended membership graphs for picture inference systems for KG.

DiffPool [8] is a DL method using a differential graph pooling technique that generates hierarchical representations of graphs. The experimental results (for DiffPool) show an average improvement in the accuracy for graph classification in the range 5% to 10% when compared to the alternative pooling methods considered [8]. However, control of the learning process is difficult given the complexity and large number of parameters in an end-to-end model. In related research, Ying et al. [8] and Lee et al. [9] have implemented the DiffPool model with experiments using the same parameters. The GNN model has demonstrated (a) the capacity to address complex hierarchical structures and the related results derived from the clustering process and (b) a reduction in the number of parameters with improved efficiency [1,8]. In considering the GNN model, Ying et al. [8] have also identified reductions in the computational cost while addressing a larger number of training models. However, the experimental results identify an unstable training issue with variable prediction accuracy [8,9]. Moreover, the identification of the appropriate number of clusters, when there are datasets with a large number of clusters, represents a significant challenge.

Context and context-awareness (which includes situational-awareness) are important considerations in future intelligent information systems (IS) where intelligent context processing with decision support (a process designed to enable personalisation and targeted service provision (TSP) [10,11] in a diverse range of domains) is an important component in context-aware cyber-physical systems (CACPS) [12,13]. “Embedded system design: embedded systems foundations of cyber-physical systems, and the Internet of Things” is introduced in [14].

Peter Marwedel [14] considers the design of such systems and identifies the opportunities in domains including (a) automotive electronics; (b) railways; (c) ships, ocean technology, and maritime systems; (d) factory automation; (e) the health sector; (f) data analytics; (g) smart environments (cities and buildings; (h) smart grids; (i) scientific experiments; (j) structured health monitoring; (k) disaster recovery; (l) robotics; (m) agriculture and breeding; (n) military applications; (o) telecommunications; and (p) consumer electronics. Additionally, Marwedel addresses the related challenges: (a) dependability and reliability; (b) safety; (c) security, privacy, and confidentiality; and (d) availability and reparability. Constraints include hard time constraints (applicable to mission-critical systems) and soft time constraints.

It is significant that intelligent CACPS (including cloud-based systems using the Internet of Things (IoT) in networked sensor-enabled systems) generally operate on large datasets [14]. Intelligent context-awareness with decision support under uncertainty has been considered in [15], and the application of rules in knowledge reasoning for inference has been addressed in [16]. Future intelligent CAPS designed to realise TSP and control will require advanced machine learning techniques to address the demands of affective computing [17,18] and machine cognition with emotive response [19] in, for example, humanoid robots [14,18]. To address these demands, we must accommodate large datasets and use advanced inference and reasoning (including fuzzy systems with linguistics and semantics using *Kansei* engineering and hedge algebras [20]) if we are to realise intelligent information processing [21].

In this paper, we propose the FPool framework, which is predicated on the basic approach adopted in DiffPool where pooling is applied directly to node representations. FPool is conceived as an approach designed to enhance data normalisation. We have evaluated FPool using popular and publicly available sensor datasets (see Section 5). In a comparative analysis (where all methods compared are implemented using the same datasets), the experimental results demonstrate improved classification and prediction performance when compared to the alternative methods considered. Moreover, FPool shows an important reduction in the training time over the DiffPool framework. The proposed FPool model is introduced in Section 4 with a discussion on evaluation and experimental results set out in Section 5.

Our contribution can be summarised as follows:

- The FPool approach implementing hierarchical pooling in a GNN to enhance classification performance for large datasets with significant reductions in training time.
- The FPool framework (see Figure 3) has been conceived and designed to reduce the number of parameters in a GNN. Specifically, a single GNN layer will learn node representation $X^{\left(l\right)}$ for all nodes in the graph with the representation used to assign nodes to clusters.

The remainder of this paper is structured as follows. Related research is considered in Section 2. Materials and methods are addressed in Section 3, where GNN, graph classification, and hierarchical pooling are introduced. The proposed approach is presented in Section 4. The basis for experimental testing and comparative analyses is set out in Section 5 with the experimental results provided in Section 5.2. Simulation results for FPool are set out in Section 5.3 with an evaluation of FPool and DiffPool presented in Section 5.4. A discussion is presented in Section 6 with open research questions introduced in Section 6.1. Concluding observations are provided in Section 7.

## 2. Related Research

Research has investigated many diverse techniques to address classification performance related to large datasets. Many advanced methods of applying DL to structured data (for example, graphs) have been proposed which focus on generalising CNN to graph data. This approach includes redefining the convolution and the downsampling (pooling) operations for graphs. In the literature, there are a number of proposed methods and techniques based on DL and the GCN concept including (a) Self-Attention Graph Pooling (SAGPool) [9], (b) inductive representation learning on large graphs (GraphSAGE) [22], (c) GNN with KG ([23]), (d) Graph Attention Networks (GAN) [24], (e) Self-Attention Generative Adversarial Networks (SAGAN) [25], DiffPool [8], (f) a Graph Isomorphism Network (GIN) [4], (g) EdgePool [1], and (h) a GCN [26] (see Section 3.1). Text categorisation as a graph classification problem has been addressed in [27].

A Self-Attention Graph Pooling (SAGPool) approach, a graph pooling method for GNN related to hierarchical graph pooling, is proposed by Lee et al. in [9]. SAGPool enables pooling with consideration of both node features and graph topology. In SAGPool, the self-attention mechanism can distinguish between the nodes that should be dropped and the nodes that should be retained [9]. SAGPool employs graph convolution to calculate attention scores and node features along with consideration of graph topology. The reported experimental results demonstrate that SAGPool realises improved graph classification performance on benchmark datasets using a reasonable number of parameters. The authors posit that SAGPool provides advantages over the alternative methods considered in [9] and is proposed as the first method to use self-attention for graph pooling with high performance.

GraphSAGE [22] is a general inductive framework designed to utilise the feature data of nodes including where such data includes text attributes. The goal for GraphSAGE is to efficiently generate node embeddings for previously unseen data. The approach enables unsupervised learning on graphs to overcome the “*Out of memory*” problem experienced by GCN. Hamilton et al. [22] have also developed other aggregation functions including *MEAN*, *SUM*, and *Long short-term memory* (LSTM) [28] which can potentially increase the diversity of GNN methods. A GNN using a knowledge graph (KG) has been proposed in [23] for recommendation systems to enhance the classification performance accuracy.

The GAT method is designed to operate on graph structured data [24]. The method uses a neural network architecture to leverage self-attentional layers with the aim of addressing issues (identified in alternative methods) based on graph convolutions or their approximations [24]. The approach applies layer stacking [24] where nodes can access neighbourhood features. The reported results posit [24] (a) the “implicit” specification of different nodes in a neighbourhood without the costly matrix operation (such as inversion) or prior knowledge of the graph structure, and (b) the realisation of an improvement in classification performance (where test graphs are unseen during training) using four established transductive and inductive graph benchmark datasets: (i) *Cora*, (ii) *Citeseer*, (iii) *Pubmed* citation network, and (iv) a *protein interaction* dataset.

Zhang et al. in [25] propose the Self-Attention Generative Adversarial Network (SAGAN) approach which enables attention-driven, long-range dependency modelling for image generation tasks. It is argued in [25] that traditional convolutional generative adversarial networks (GAN) generate high-resolution details as a function of only spatially local points in lower-resolution feature maps. Moreover, SAGAN provides a basis upon which details can be generated using cues from all feature locations given that the discriminator can check for highly detailed features in distant portions of the image which are consistent with each other. The reported experimental results show that SAGAN improves on the best published Inception score (36.8) with a score of (52.52) along with a reduction in the Fréchet Inception distance from (27.62) to (18.65) for the ‘ImageNet’ dataset. From a visualisation perspective, the authors argue that based on attention layers the generator can leverage neighbourhoods that correspond to object shapes rather than local regions of fixed shape.

Ying et al. in [8] proposed the use of the DiffPool approach to address the requirements of graph representational learning based on effective learned node embeddings. An overview of the DiffPool method is provided in Section 1.

The Graph Isomorphism Network (GIN) [4] represents the node features on the graph as multisets with possibly repeating elements by aggregated function. Moreover, GIN is proven to be as powerful as the Weisfeiler–Lehman test. GIN differs from DiffPool in that DiffPool uses hierarchical pooling throughout sequential assignment layers, whereas GIN can be considered as a global pooling architecture in which the graph embedding is the mean of its node embeddings.

GNN focus on improving convolutional layers; however, limited attention is applied in the development of graph pooling layers [1]. Pooling layers can provide an effective basis upon which GNN can reason over abstracted groups of nodes instead of single nodes, thus increasing their generalisation potential [1]. To address this issue, Diehl et al. [1] propose the *EdgePool* method in which a graph pooling layer relies on the notion of edge contraction; *EdgePool* applies the learning of a localised and sparse pooling transform. Evaluation using four datasets found that improved performance was achieved for three largest datasets. Moreover, Diehl et al. show that EdgePool can be integrated in existing GNN architectures without adding any additional losses or regularisation.

In future intelligent IS, affective computing and the identification of emotional response will be an increasingly an import feature implemented using text analysis [20]. Rousseau et al. in [27] has considered text categorisation as a graph classification problem where each document is represented as a graph-of-words instead of the historical n-gram bag-of-words. By utilising the power of graph structures, the graph-of-words captures the word inversion with subset matching (e.g., article about news vs. news article) while the bag-of-words fails to enable word inversion and subset matching.

Generalising the convolution operation for graphs has been shown to provide improved levels of performance and accordingly has been widely used. However, (a) the method of applying downsampling to graphs remains a challenge with significant room for improvement, and (b) while alternative proposed methods considered achieve good results, the current GNN methods are “inherently flat and do not learn hierarchical representations of graphs” (this limitation can be an issue where the goal is to predict the label associated for an entire graph).

DiffPool introduces a differential graph pooling module that can generate hierarchical representations of graphs and can be combined with various graph neural network architectures in an end-to-end fashion [8]. DiffPool is a deep learning approach which learns a differential soft cluster assignment for nodes at each layer of a deep graph neural network with nodes mapped to sets of clusters. There is, however, an issue (see Section 1) where control of the learning process is difficult given the complexity and large number of parameters in an end-to-end model.

While GIN, DiffPool and SAGPool perform pooling on graph nodes, SAGPool [1] proposes a new pooling layer which performs edge contractions. The experimental results show that EdgePool achieves high prediction accuracy on several benchmark datasets. However: (a) EdgePool always pools roughly half of the total nodes, and (b) the experimental results show that EdgePool fails to capture the hierarchical structures of some datasets.

### Summary

For the classification of knowledge graphs, a number of graph classification methods have been considered to address graph classification performance related to large datasets. Many methods of applying DL to structured data (for example, graphs) focus on generalising CNN to graph data; this approach includes redefining the convolution and the downsampling (or pooling) operations on graphs. Other approaches have implemented Hierarchical Pooling (HP).

Text categorisation as a graph classification problem [27] represents an interesting approach which may be applied to address the requirements of text analysis in many domains. Applying such methods presents a potentially useful approach for analysing text in intelligent context-aware systems designed to enable affective computing and emotion recognition. Such an approach is applicable to sensor-driven autonomous robots designed to achieve coverage path-planning (CPP) as discussed in [29].

To address the issues identified in our review of the related research we propose our novel FPool framework which is predicated on the DiffPool method. While providing an interesting approach, DiffPool has suffered from overfitting while training an end-to-end model.

The study presented in this paper has investigated adjustments in parameters along with the related training process completion. For example, in the alternative methods considered, the graph nodes are pooled to very few clusters (they only include two clusters). In pooling, with a reduction in the number of clusters and nodes, there is a commensurate reduction in the training parameters. Given the reduction in the number of clusters, many clusters (present in the datasets) may not be used in the training process leading to significant redundant clusters and/or redundant parameters.

The proposed FPool framework is introduced in Section 4 with the materials and methods provided in Section 3. In summary, (a) in FPool the pooling process is performed directly on node embedding which reduces the number of parameters, and (b) to accelerate the training and improve the generalisation for the novel FPool techniques and methods developed and tested. In FPool, the node features will have the zero mean and unit variance properties as discussed in [30].

While our study has researched and addressed many issues, we have identified open research questions (see Section 6.1) which form the basis for future directions for research.

## 3. Material and Methods

In this section, we introduce the materials and methods used in this study, namely, GNN (see Section 3.1), GCN (see Section 3.2), and HP (see Section 3.4. The proposed model is introduced in Section 4.

### 3.1. Graph Neural Networks

Let G(V,E) be a graph, each node v∈V has a feature vector $x_{v}\in R^{d}$. A GNN uses the graph structure and the node features to learn a vector representation $h_{v}$ for each node. Recent GNN methods follow the message-passing mechanism where the vector representation of each node is iteratively updated by aggregating the hidden representations of neighbour nodes [9,26]. Following completion of the *k* iteration, the vector representation of *v* holds the information of the k-hop network where *v* is a centre vertex. For instance, at iteration *k*, GNN perform these functions, given by Equations (1) and (2):

(1) $$a_{v}^{\left(k\right)}=AGGREGATE^{\left(k\right)}\left(\left\{h_{u}^{\left(k-1\right)}:u\in N\left(v\right)\right\}\right)$$

(2) $$h_{v}^{\left(k\right)}=COMBINE^{\left(k\right)}\left(h_{u}^{\left(k-1\right)},a_{v}^{\left(k\right)}\right)$$

where $a_{v}^{\left(k\right)}$ and $h_{v}^{\left(k\right)}$ represent vectors of N(v) and *v* at iteration k, respectively. N(v) indicates the set of neighbour(s) of node *v* where the set of neighbour(s) of node *v* is composed of direct neighbour(s). For initialisation, the representation of node *v* is completed before forwarding to the first AGGREGATE function $h_{v}^{\left(0\right)}=x_{v}$.

There are a number of AGGREGATE and COMBINE functions. For example, GraphSAGE-MAX [22] uses the AGGREGATE function as given by Equation (3):

(3) $$a_{v}^{\left(k\right)}=MAX\left(\left\{ReLU\left(W\cdot h_{u}^{\left(k-1\right)}\right),\forall u\in N\left(v\right)\right\}\right)$$

where *W* is a learning matrix parameter and MAX is the maximum element-wise function. The COMBINE function at Equation (2) represents a vector concatenation (or the summation) of the element-wise function followed by a mapping matrix $W\cdot [h_{v}^{\left(k-1\right)},\;a_{v}^{\left(k\right)}]$.

A further relevant example of a GCN where the mean element-wise is implemented is shown in [26]. The AGGREGATE and COMBINE functions are shown in Equation (4):

(4) $$h_{v}^{\left(k\right)}=ReLU\left(W\cdot MEAN\left\{ReLU\left(W\cdot h_{u}^{\left(k-1\right)}:\forall u\in N\left(v\right)\right)\right\}\right)$$

**Figure 1:**
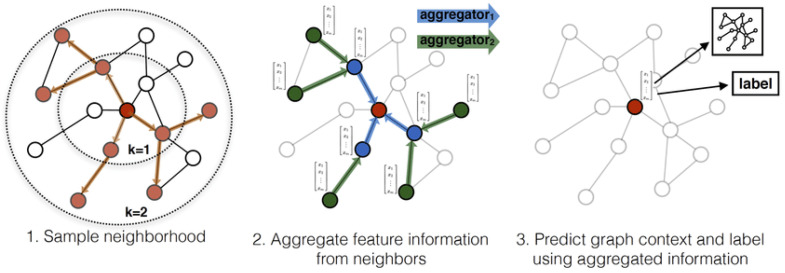
Illustration of a general GNN inductive framework on a specific node (the red node) source: [22]. The figure illustrates the iterative process of node assignment in the simple neighbourhood with the aggregated feature information from neighbours. The predicted graph context and label (using the aggregated information in step 2) is shown in this Figure.

Figure 1 illustrates the GNN process on a specific (red) node showing (1) a simple neighbourhood, (2) aggregate feature information from neighbours, and (3) the prediction of the graph context and label using aggregated information.

In the initial stage, termed the neighbourhood sampling stage, a number of neighbour nodes are selected. For large graphs, neighbourhood sampling is essential to address the memory consumption issue when a large number of nodes with large number of GNN layers easily leads to the `out of memory’ error. Following the sampling of neighbour nodes, the AGGREGATE and COMBINE functions are implemented. Thus, the hidden representation of a node is forwarded to downstream tasks such as node classification and clustering.

### 3.2. A Formal Description of Graph Neural Networks

Formally, a GCN is a neural network that operates on graphs. Given a graph $G=\left(V,\;E\right)$, a GCN takes as input an input feature matrix $X\in R^{n\times d}$ where *n* is the number of nodes and *d* is the number of input features for each node and an n×n matrix representation of the graph structure such as the adjacency matrix *A* of *G*. A hidden layer can be written as $H^{\left(i\right)}=f\left(H^{\left(i-1\right)},A\right)$, where $H^{\left(0\right)}=X$ and *f* is a propagation function. Each layer $H^{\left(i\right)}$ corresponds to a $n\times d_{i}$ feature matrix where each row is a feature representation of a node. At each layer, these features are aggregated to form the features for the next layer using the propagation rule *f*. Features become increasingly more abstract at each consecutive layer, and with this framework variants of GCN differ only in the choice of propagation rule *f*.

Specifically, the GCN approach is inspired by the notion of CNN for image processing. CNN aggregates the adjacent pixels of the current pixel to extract local features, such as shapes and backgrounds, of an image. When considering graphs, while image processing operates on pixels, the GCN operates on node features. For each vertex on the graph, the GCN approach aggregates the features of neighbour vertices and then generates the hidden representations for that vertex.

### 3.3. Graph Classification

Graph classification is a crucial task where the aim is to identify the labels for each graph in large sensor data sets. Consider, for instance, chemistry where the prediction of chemical properties (e.g., toxicity of molecules) is crucial in medical research. Moreover, graph classification is applied to biomedical networks to predict protein functions [31] where (i) each graph represents exactly one protein and (ii) nodes indicate secondary structure elements (helices, sheets, and turns). Edges connect nodes if those are neighbours along the amino acids and neighbours in the space within the protein structure.

In a graph there are two main classification tasks including (i) on the node-level, and (ii) on the graph level.

1. For **node** classification, each node *v* has an associated label $y_{v}$ and the goal is to learn a representation vector $h_{v}$ that could be used to predict the label $y_{v}$ by using a function *f*, $y=f(h_{v})$.
2. For **graph** classification, given a set of graphs $G_{1},G_{2},\ldots G_{n}$ and their labels $y_{1},y_{2},\ldots y_{n}$, instead of learning $h_{v}$ for each node, the model aims to learn the representation vector $h_{G}$ for the whole graph so that $h_{G}$ helps to predict the label of graph, $y_{G}=g\left(h_{G}\right)$.

### 3.4. Hierarchical Pooling

Conventional approaches (for example, see Xu et al. [4] and Duvenaud et al. [5]) do not capture the hierarchical properties of graphs while all the node embeddings are globally pooled. The embedding of a graph is therefore similar to a virtual node that connects to all the nodes of the graph and such common approaches have not addressed the need to learn the natural structures of many real-world graphs.

Ying et al. [8] proposed the *DiffPool* approach, a differentiable graph pooling method which learns a cluster assignment matrix in an end-to-end fashion. The key motivation for *DiffPool* is to induce learning to enable nodes to be assigned to clusters at layer *l* by using the embeddings generated from the GNN layer at layer $\left[l-1\right]$.

We have denoted $n_{l}$ as the number of nodes at layer *l*, l≤L, where l=0 is the number of nodes of the original graph *G* and *L* is the maximum number of pooling layers. $S^{\left(l+1\right)}\in R^{n_{l}\times n_{l+1}}$ denotes the assignment matrix at layer l+1, and $GNN_{l}$ represents the l−th GNN layer. Each GNN layer or module contains *K* message-passing iterations; this means that each GNN module will repeat the Equations (1) and (2) *K* times. We use the notations $GNN_{l,pool}$ and $GNN_{l,embed}$ to indicate two kinds of GNN module used in DiffPool. $GNN_{l,pool}$ is for pooling the graph and $GNN_{l,embed}$ is for learning node embeddings. Equation (5) expresses the learning node embeddings $Z^{\left(l\right)}\in R^{n_{l-1}\times d_{l}}$ for all nodes in the graph, given $l=\bar{1,L}$.

(5) $$Z^{\left(l\right)}=GNN_{l,embed}\left(A^{\left(l-1\right)},X^{\left(l-1\right)}\right)$$

To generate the assignment matrix, DiffPool employs Equation (6).

(6) $$S^{\left(l\right)}=softmax\left(GNN_{l,pool}\left(A^{\left(l-1\right)},X^{\left(l-1\right)}\right)\right)$$

Therefore, $S_{ij}^{\left(l\right)}$ (the value at row *i*, column *j* of the 2-dimensional matrix $S^{\left(l\right)}$) contains the probability of node *i* at layer *l* assigned to cluster *j* at the next layer. After learning the node embedding matrix $\left(Z^{\left(l\right)}\right)$ at layer *l*, the node features matrix $X^{\left(l\right)}$ and the adjacency matrix $A^{\left(l\right)}$ of the new graph at layer *l* is expressed by Equation (7) and (8):

(7) $$X^{\left(l\right)}=S^{(l)T}Z^{\left(l\right)}$$

(8) $$A^{\left(l\right)}=S^{(l)T}A^{\left(l-1\right)}S^{\left(l\right)}$$

**Figure 2:**
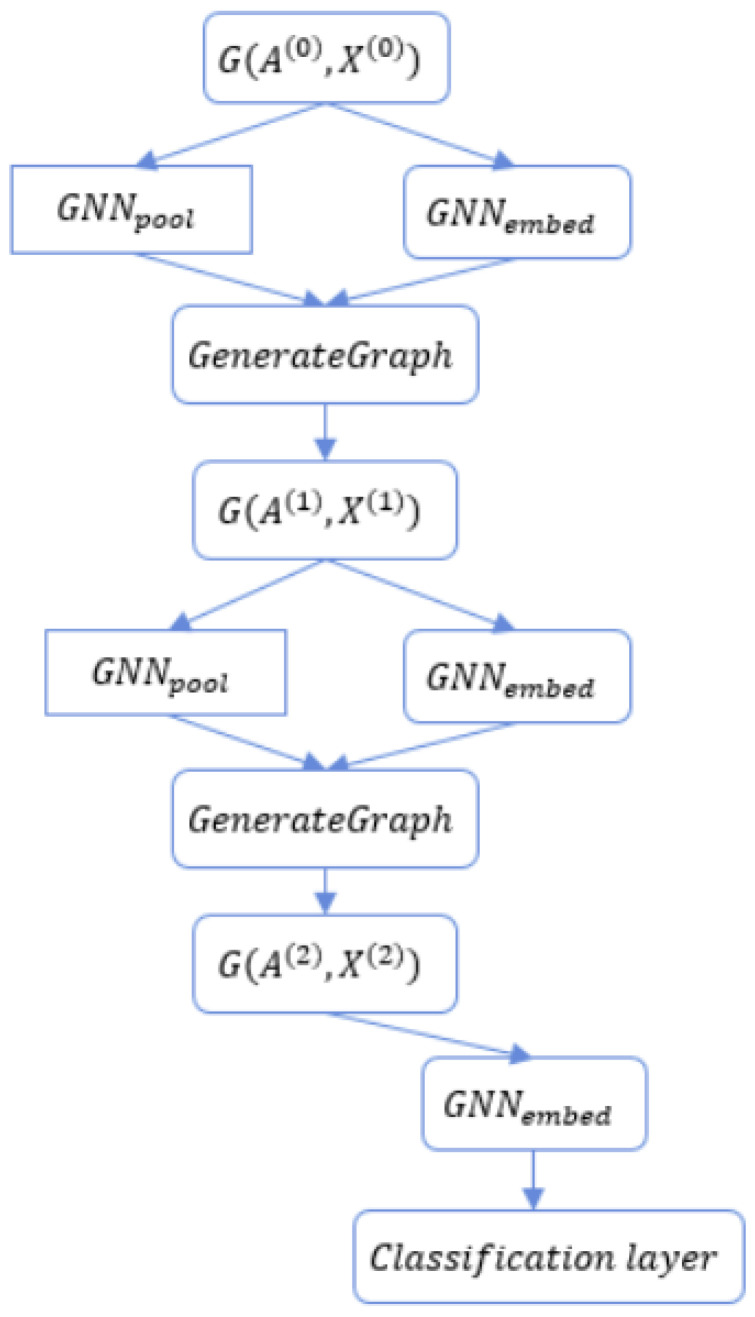
An overview of the *DiffPool* framework with 2 pooling layers where the input is a graph $G(A^{\left(0\right)},X^{\left(0\right)})$ and the output is the predicted label for that graph at the classification layer.

The *DiffPool* framework is illustrated at Figure 2. Given an input graph $G(A^{\left(0\right)},X^{\left(0\right)})$, the adjacency matrix and the node features are forwarded to two separated GNN: $GNN_{1,pool}$ and $GNN_{1,embed}$. In the l−th ‘GenerateGraph’ stage, the new graph is generated given the output of $\left(GNN_{l,embed}\right)$ (denoted at $Z^{\left(l\right)}$) and the output of $GNN_{l,pool}$ (denoted at $S^{\left(l\right)}$). Equations (7) and (8) are then implemented.

To predict the label for each graph, the final layer of the DiffPool framework would be the classification layer with a softmax function. However, it is difficult to train the DiffPool framework using only the gradient from the classification layer. Therefore, Ying et al. in [8] proposes two alternative loss functions: (i) the *link prediction* loss and (ii) the *entropy* loss. The link prediction loss aims to pool nearby nodes at each layer *l*, $l=\bar{1,L}$; that loss function is expressed by Equation (9).

(9) $$L_{LP}={∥A^{\left(l\right)},S^{\left(l\right)}S^{(l)T}∥}_{F}$$

where ${∥.∥}_{F}$ is the Frobenius norm (note: each node assigns completely to a cluster). Moreover, the entropy loss is assigned to a vector for each node in a one-hot vector. The entropy loss used is given by Equation (10).

(10) $$L_{E}=\frac{1}{n}\sum_{i=1}^{n}H\left(s_{i}\right)$$

where *H* denotes the entropy function and $s_{i}$ is the assignment vector for node *i*. Therefore, the whole framework is trained by using the combination of these loss functions.

## 4. The Proposed Model

**Figure 3:**
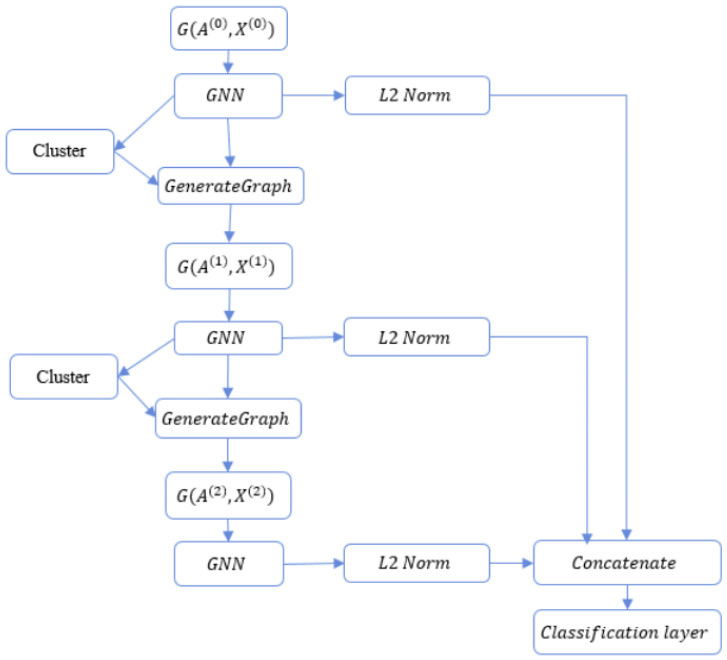
The three-layer FPool framework (the proposed FPool model is introduced in Section 4). The input is a graph $G(A^{\left(0\right)},X^{\left(0\right)})$ and the output (in the classification layer) is the predicted label for that graph. The evaluation and experimental results are set out in Section 5 with the simulation results presented in Figure 4.

In this section, we introduce the proposed FPool framework (see Figure 3) which is based on the DiffPool framework (see Figure 2). The methods and processes introduced in the FPool framework are set out in Section 3. FPool has been conceived and designed to (a) realise improvements to the DiffPool framework and (b) address issues identified in Section 2. FPool is a novel approach designed to incorporate significant improvements which are:

- FPool uses only one type of GNN ($GNN_{merged}$) to learn the node representation and node assignment to clusters, whereas DiffPool uses two types of GNN ($GNN_{pool}$ and $GNN_{embed}$). Therefore, when compared to DiffPool, FPool employs a different graph pooling process and the number of GNN parameters is reduced by half (50%).
- In the output graph embedding process, every layer is concatenated prior to forwarding to the classification layer (to predict the graph label). The output embedding process is inspired by the “skip-connection” idea proposed in the Resnet architecture introduced in [32].

FPool implements a pooling and embedding stage where learning is implemented differently to DiffPool. In FPool, given an input graph $G(A^{\left(l\right)},\;X^{\left(l\right)})$, the adjacency matrix $A^{\left(l\right)}$ and the node feature matrix $X^{\left(l\right)}$ are sent to the $GNN_{l,merged}$ to learn the node embedding. Thus, the learned node embeddings are used in the assignment of nodes to clusters.

In FPool, the pooling process is performed directly on the node embedding, which reduces the number of parameters. To accelerate the training and improve the generalisation of the model, we have added (i) normalisation techniques consisting of *L2 normalisation* applied to node representations and (ii) *centring* and *scaling* used for node features in preparation for the training process. As a result, the node features will have the zero mean and unit variance properties as discussed in [30].

The proposed approach has been conceived and designed to reduce the number of parameters in the GNN. Specifically, a single GNN layer will learn node representations $X^{\left(l\right)}$ for all nodes in the graph with the representations used to assign nodes into clusters. The FPool model integrates both the $GNN_{pool}$ and $GNN_{embed}$ methods as shown in Figure 2. FPool has been conceived to enable the merging of these models by reducing the number of parameters of the GNN. Specifically, a single $GNN_{l,merged}$ layer will learn node representations $X^{\left(l\right)}$ for all nodes in the graph and the representations are thus used to assign nodes into clusters. The FPool framework is illustrated in Figure 3. In the *FPool* framework, computing the node embeddings $Z^{\left(l\right)}\in R^{n_{l-1}\times d_{l}}$ and matrix $S^{\left(l\right)}\in R^{n_{l-1}\times n_{l}}$ at layer *l* with $l=\bar{1,L}$ is given by Equations (11) and (12):

(11) $$Z^{\left(l\right)}=GNN_{l,merged}\left(A^{\left(l-1\right)},X^{\left(l-1\right)}\right)$$

(12) $$S^{\left(l\right)}=softmax\left(Z^{\left(l\right)}.W^{\left(l\right)}+B^{\left(l\right)}\right)$$

where $W^{\left(l\right)}\in R^{d_{l}\times n_{l}}$ is a weight matrix, $B^{\left(l\right)}\in R^{n_{l}}$ denotes the bias matrix, and the $\left(softmax\right)$ function is applied to every row of the matrix. Equations (11) and (12) are equivalent to the GNN and cluster stages in the *FPool* framework. In the GenerateGraph stage, similar to DiffPool, Equations (7) and (8) are used given the input node embeddings $Z^{\left(l\right)}$ and the assignment matrix $S^{\left(l\right)}$ from Equations (11) and (12), respectively. Therefore, the new pooled graph $G(A^{\left(l+1\right)},X^{\left(l+1\right)})$ is generated.

An overview of related methods is presented in Section 2 with a comparative analysis provided Section 5. Table 2 provides results for the classification accuracy and Table 3 sets out in tabulated form the training time analysis. In considering the related research, the alternative methods often increase the number of GNN layers from two to six. As a result of increasing the number of layers in a GNN, there may be a failure to capture additional information [4,8]. To address this issue in the FPool method, the graph representation is the combination of all of the $\left(L\right)$ GNN layers (instead of using only the output of the GNN from the final layer). As shown in Figure 3 with three out-edges from the GNN blocks, the local node embedding $Z^{\left(l\right)}$ at all layers are normalised and concatenated to generate graph embedding $h_{G}$ prior to forwarding to the classification layer. Specifically, the graph embedding $h_{G}$ is computed as shown in Equations (13) and (14), where $Z_{F}^{\left(l\right)}$ is the L2−normalised node embedding matrix in the graph at layer *l*. Given that $z_{i}^{\left(l\right)}$ and $z_{i,F}^{\left(l\right)}$ are the node embedding of node *i* at layer l and its normalised version, $z_{i}^{\left(l\right)}$ and $z_{i,F}^{\left(l\right)}$ are equivalent to one row of $Z^{\left(l\right)}$ and $Z_{F}^{\left(l\right)}$, respectively. Therefore, $z_{i,F}^{\left(l\right)}$ is computed as shown in Equations (13) and (14):

(13) $$Z_{F}^{\left(l\right)}=Z^{\left(l\right)}/{∥Z^{\left(l\right)}∥}_{F},l=\bar{1,L}$$

(14) $$h_{G}=\left[MEAN\left(Z_{F}^{\left(1\right)}\right),\;\ldots \;MEAN\left(Z_{F}^{\left(L\right)}\right)\right]$$

Equations (13) and (14) are equivalent to the *L2 Norm* and the *Concatenation* stages in Figure 3. By standardising node embeddings, the training process becomes more stable. The MEAN represents an element-wise mean function and [.] denotes the vector concatenation function. Therefore, $h_{G}$ has the size $d_{1}+d_{2}+\ldots +d_{L}$, where $d_{1},d_{2},\ldots ,d_{L}$ is the size of the node representation at layers 1,2,…L, respectively.

The notion of vector concatenation is inspired by the *Residual block* on the *Resnet architecture* in computer vision [32]. Optimisation of deep networks is challenging and increasing network depth may not lead to better performance due to the ‘vanishing gradient’ problem. Moreover, stacking more layers onto the network may result in `performance saturation’ with reduced performance [33,34]. The study presented in [32] proposes `short-cut connections’ (an approach which skips one or more layers) where the gradient from upper layers could ‘flow directly’ to any earlier layers.

In the initialisation step, the node features matrix $X^{\left(0\right)}\in R^{n_{0}\times d_{0}}$ is normalised to have zero mean and unit variance. This step is crucial as it leads the machine learning model to converge faster and perform better [30]. The normalisation equation for node features matrix *X* at column *j* is represented as given by Equation (15) where $\mu_{j}$ and $\sigma_{j}$ denotes the mean and standard variance values of *X* on column *j*, respectively.

(15) $$X_{norm,j}^{\left(0\right)}=\frac{X_{j}^{\left(0\right)}-\mu_{j}}{\sigma_{j}},j=\bar{1,d_{0}}$$

## 5. Experimental Testing and Evaluation

To evaluate the performance of the proposed model, we have conducted a comparative analysis where we compare the relative performance of FPool) with the alternative methods considered see Tables 2 and 3). Implementation for all the methods evaluated has used the same datasets under the same testing environment. In the experimental testing we have used several popular and publicly available data sets for the evaluation with graph classification tasks; these sensor datasets are publicly available at http://graphkernels.cs.tu-dortmund.de (accessed on 6 September 2021) [35]. The benchmark sensor data sets are described in Table 1 where the identification, reference, and descriptive information is provided.

Each dataset is a set of graphs where each graph has an associated label with each node on the graph having its attribute and label. The node attribute can have various dimensions on different datasets while the node label has only 1-dimension and, for a dataset where the node attribute is not available, we denote the dimension as $\left\{0\right\}$. Because our task is graph-level classification, it is not necessary to discriminate between the node attribute and label. Therefore, the node feature vector is initialised with the combination of the attribute and label. The datasets used in our comparative analysis are shown in Table 1 with a brief introduction to the datasets provided as follows.

1. Mutag: [35]: the dataset consists of 188 graphs equivalent to 188 chemical compounds. These graphs are divided into two classes based on their *mutagenic* sensors effect on a bacterium.
2. Enzymes [31,36]: a biological dataset for enzymes. The sensor dataset contains 600 enzymes with six associated classes which represent the characteristics of enzymes. Each graph represents exactly one protein, nodes indicate the secondary structure (SSE) in protein, and there exists a connection between two vertices if they are neighbours in the amino-acid or on the 3-D space.
3. IMDB-Binary [37]: a social networks dataset in which each graph is equivalent to an ego-network where nodes represent actors, edges denote two actors collaborating in a film. Each graph is derived from a pre-specified genre of film.
4. D&D [38]: a sensor dataset of protein structures which includes 1178 graphs. Nodes indicate amino acids and edges denote that two nodes are close to each other on 3-D space.

In a GNN, each vertex in the input graph must have an associated feature vector. Therefore, for graphs without a node feature matrix, we initialise it as a vector of constant values $\left(x_{v}\;=\;\left[1,1\right],\;\forall \;v\;\in \;V\right)$. For graphs which contain node labels or node attributes (or both) the feature vector for each vertex is then a concatenation of node attribute vector and node label vector.

We have selected several methods as a comparison baseline (including DiffPool because FPool is predicated on, and is designed to directly improve, the DiffPool approach). The methods implemented in the comparative analysis are shown in Table 2 and Table 3. Each of the alternative methods considered uses a different pooling architecture. While DiffPool and SAGPool perform hierarchical pooling on nodes, EdgePool proposes a new pooling layer which reduces the size of the graph based on edges. As discussed in [4], GIN uses a global pooling architecture where the model is simpler and (as for any mean aggregator) to facilitate graph embedding $X_{1}$ and $X_{2}$ are mapped to the same embedding because the aggregator simply takes averages over individual element features [4]. Thus, the mean captures the distribution (proportions) of elements in a multiset, but not the exact multiset [4].

Implementation (for all methods in the comparative analysis) has been achieved using *Pytorch Geometric library* (see [39]). Pytorch Geometric is a Python library which supports many types of GNN (with some minor editing) along with many processed datasets (including all of the data sets used in our experiments). For FPool K=3 relates to message-passing iterations of GraphSAGE-MEAN for each GNN module in Figure 3. The number of hidden units is 64, with the size of the hidden representation vector being $h_{v}^{\left(k\right)}$ and $k=\bar{1,K}$. The number of clusters is 25 for both the first and the second pooling layers on the *Mutag*, *Enzymes*, and *IMDB-Binary* datasets. On the D & D dataset, the number of clusters is larger and is set to 125. For the final classification layer, both the *DiffPool* and *FPool* use the same architecture:

1. Linear: embedding_size, hidden_size
2. ReLU - Linear: hidden_size, n_classes
3. Log Softmax: where embedding_size is the size of $h_{G}$, hidden_size is the number of hidden units and n_classes indicates the number of classes to be classified

### 5.1. Loss Functions

Algorithm development aims to minimise inaccuracy in classifying models and the loss function is a measure of the algorithm’s performance [40]. Fundamentally, a *loss function* is a relatively simple concept which measures the ability of an algorithm to model a dataset where the numerical output from a loss function will be higher relative to the degree of inaccuracy [41]. It is beyond the scope of this paper to provide a detailed discussion on the topic of loss functions (for a detailed exposition on the nature of loss functions with consideration of the differing types with proofs see in [40,42,43,44]).

As discussed in Section 6.1, the loss functions used in Fpool have been inherited from DiffPool because FPool is based on the DiffPool framework, namely, *classification loss*, *link prediction loss*, and *entropy loss*. While *classification loss* and *entropy loss* are generally known and understood, *link prediction loss* is less well recognised.

*Link prediction loss* assists in the identification of edges that are likely to arise in the future (always assuming they do not currently exist). For link prediction, the resolution of entities employs the network structure and attribute data to link nodes representing the same individual. Link prediction may assess ranking related to the expected relationships that exist between candidate nodes and links. A typical application may attempt to predict papers (an author may for example cite, read, or write) based on (a) previous publication history and/or (b) current research trends related to similar topics. For example, GraphSAGE [22] has built a model that predicts citation links in the *Cora* dataset [45].

### 5.2. Experimental Results on Classification Accuracy and Training Time

In the experimental testing and evaluation, the data is separated into three sets: (i) a *training* set, (ii) a *validation* set, and (iii) a *test* set; the relative proportions are 8:1:1 (i.e., 80%, 10%, and 10%), respectively. The validation set is evaluated during training after each epoch. When the training process completes, we identify the optimal model (which produces the highest classification accuracy on the validation set to evaluate on the test set). To avoid bias, and ensure a fair comparative analysis, we have applied the same approach to implementation for all the methods compared including using the same training, validation, and test sets at each time of running. We evaluate the accuracy on each data set using 10 running iterations; therefore, there are 10 different combinations of the training, validation, and testing.

The results derived from our experimental testing identify the *mean value* and standard variance to measure the accuracy as shown in Equation (16):

(16) $$Acc=\frac{1}{N}\sum_{i=1}^{N}\left(y_{pred,i}==y_{true,i}\right)$$

where $y_{true,i}$ is the actual class of graph *i*, $y_{pred,i}$ is the predicted class for graph *i*, and *N* denotes the number of graphs in a test set.

As shown in Table 2 and Table 3, we have calculated (i) the results for the classification accuracy on the test set (see Table 2) and (ii) the training time (see Table 3). The two experiments indicate the performance of the different methods compared. the methods compared and the datasets used are shown in Table 2 (the classification performance) and Table 3 (the training time), respectively.

The training time shows the computation cost, while the classification accuracy on test set illustrates how well the model captures the structure of the graph. FPool has demonstrated a significant improvement in the classification performance when compared to the other baseline methods. For the IMDB-Binary dataset, GIN shows a shorter training time when compared to the other methods evaluated with a relatively small improvement in performance results in the range 0.5% to 1.2%. However, with a single exception (the training time for the IMDB-Binary dataset), overall FPool demonstrates a significantly improved performance over the other methods evaluated. In summary:

- GIN uses a very simple architecture and it outperforms the other methods evaluated on the training time experiment. However, it suffers from an inability to capture the hierarchical structure of many real-world datasets as shown in Table 2 with lower accuracy specifically on molecular datasets: D & D, Mutag, and ENZYMES.
- The training time for FPool is faster than DiffPool due to the reduction in the number of parameters resulting from the merging of the $GNN_{embed}$ and $GNN_{pooling}$ processes into a single process.
- EdgePool, by contracting edges, has shown the worst training time, as evidenced in the ENZYMES, IMDB-BINARY and D & D datasets.
- Compared with SAGPool (which also performs hierarchical pooling on nodes), the training time for FPool is higher in the small MUTAG dataset with the different of 0.005. However, FPool is faster than SAGPool with the increasing size of the dataset.
- The training time for FPool is faster than SAGPool on the IMDB-BINARY dataset with an improvement of 0.073 s per epoch.
- The training time for FPool is better for the D & D dataset with a value of 0.365 s per epoch.

The overall result shows that FPool scales better than the other baseline methods evaluated.

### 5.3. Simulation Results for FPool Node Clustering

**Figure 4:**
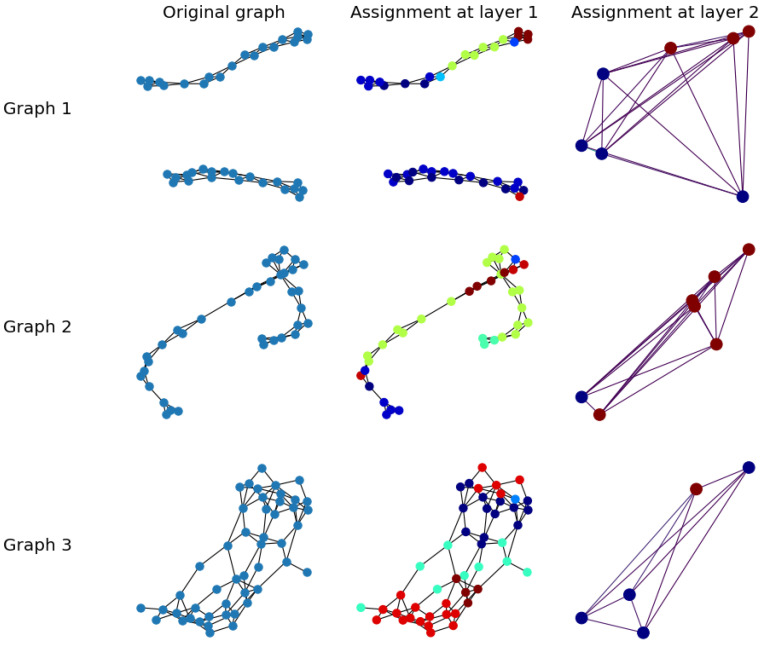
FPool visualisation of node assignment with two pooling layers on 3 example graphs from the ENZYMES dataset. Shown is graph 1, graph 2, and graph 3 with the results from the simulation for the: original graph, layer 1 assignment, and layer 2 assignment. The FPool model is introduced in Section 4 with the three layer FPool framework shown in Figure 3.

Figure 4 illustrates the hierarchical cluster assignment of FPool with two pooling layers and three example graphs taken from the ENZYMES database. Node clustering colours indicate *cluster* with *edge* colours indicating the edge weights. In the assignment process, matrices $S^{\left(l\right)}$ are real-value matrices; therefore, the generated graph is a complete weighted graph. Because of the entropy loss function, it is expected that each node will be assigned to only one cluster; therefore, in this visualisation, each node is allocated to the cluster with the highest classification value. In Figure 4, while the number of clusters is set to 25, many clusters are empty; FPool has been automatically trained to allocate nodes to the appropriate (highest value) clusters.

### 5.4. Evaluation of FPool and DiffPool Hierarchical Structures

**Figure 5:**
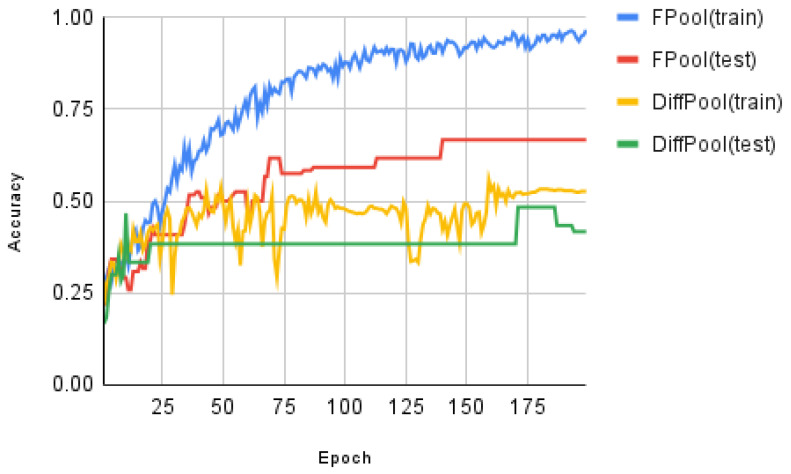
The training and test accuracy on ENZYMES dataset for: FPool(training), FPool testing), Diffpool (training), and Diffpool (testing). The X axis shows the epochs and the Y axis shows the quantitative measure of the classification accuracy.

**Figure 6:**
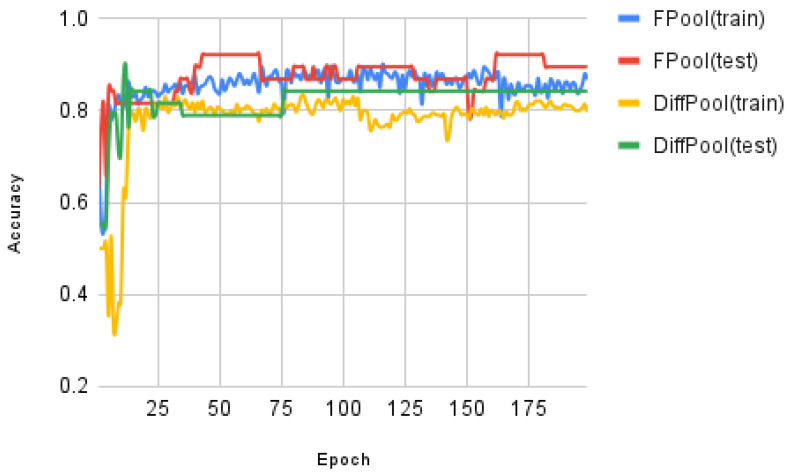
The training and test accuracy on MUTAG dataset for: FPool(training), FPool testing), Diffpool (training), and Diffpool (testing). The X axis shows the epochs and the Y axis shows the quantitative measure of the classification accuracy.

In the experimental testing, we have evaluated the training curves for both FPool and DiffPool using the ENZYMES and MUTAG datasets. The results of the evaluation are shown in Figure 5 (the training and test accuracy on ENZYMES dataset versus training epoch) and Figure 6 (the training and test accuracy on the MUTAG dataset versus training epoch). Figure 5 and Figure 6 show the results for the adaptation in the training process.

Recall that in our experimental testing and evaluation the data are separated into three sets: (i) a *training* set, (ii) a *validation* set, and (iii) a test set; the relative proportions are 8:1:1 (i.e., 80%, 10%, and 10%), respectively. The test accuracy is calculated on the current best model on the *validation set*; therefore, in theory, the trend line is usually on an up-trend. The results demonstrate that FPool provides improved performance over DiffPool in terms of graph classifications.

In testing the relative accuracy of FPool and DiffPool, experimental results show that FPool produces consistently better training accuracy results for graph classification for both the ENZYMES and MUTAG datasets. The training accuracy of FPool is higher than DiffPool for very early epoch(s) for both the ENZYMES and MUTAG datasets; this demonstrates that FPool captures the hierarchical structure faster than DiffPool though there remains an overfitting problem in the approaches because of less training data.

## 6. Discussion

In this paper, we have considered knowledge graphs in a diverse range of domains along with GNN which have been shown to enable improvements in node classification and prediction when applied to graph representation with learning node embedding to effectively represent hierarchical properties of graphs. Traditional graph classification methods are based on GNN. However, such methods generally fail to learn the hierarchical representation of graphs. Two-dimensional graphs are inherently flat and only propagate information across edges of graphs, the result is a failure to capture the hierarchical information.

Current methods have used deep learning using a differentiable graph pooling technique that generates hierarchical representations of graphs; however, control of the learning process is difficult given the complexity and large number of parameters in an end-to-end model. To address this difficulty, in this paper we propose an novel approach termed FPool which is predicated on the basic approach adopted in DiffPool (where pooling is applied directly to node representations). FPool implements that are newly designed to enhance data normalisation.

We have evaluated FPool using a number of sensor datasets (see Table 1). Experimental results demonstrate (i) improved classification and prediction performance when compared to alternative methods and (ii) significant reductions in the training time. The evaluation and experimental results derived from the comparative analysis and experimental testing are set out in Section 5, with results presented in tabulated form in Table 2 and Table 3. The comparative analysis has been conducted using four publicly available datasets, details of the datasets may be found in Section 5 with the details of the datasets shown in tabulated form in Table 1 where the number of graphs and classes are shown with the node attribute dimension and where code labels are included.

Considering future IS, there is a clear need to address the inherent complexity of context and context-aware systems with decision support to enable personalisation and targeted service provision (TSP). Moreover, the development of artificial intelligence (AI) systems and machine learning (ML) (including machine cognition) demands future intelligent IS capable of accommodating advanced learning techniques and large datasets to address the demands of *affective computing* and the realisation of emotional response, using advanced inference and reasoning to realise intelligent information processing. Addressing these requirements will call for many AI and ML techniques including the techniques discussed in this paper.

Considered from a practical managerial significance perspective (real-world practical application) we argue that the FPool framework offers the potential for implementation in real-world IS (including where intelligent IS are implemented in CACPS) which generally operate on large datasets. As discussed in Section 2, Rousseau et al. [27] has addressed text categorisation as a graph classification problem and has shown that documents can be represented as a graph-of-words instead of the historical n-gram bag-of-words. Text classification forms an important role in many context-aware systems where the identification of emotion and emotive response is a required process. Marwedel [14] has demonstrated the diverse range of domains to which embedded systems foundations of cyber-physical systems, and the Internet of Things applies along with the opportunities and challenges.

### 6.1. Open Research Questions

We have trained FPool and DiffPool using three loss functions: (i) classification loss, (ii) link prediction loss, and (iii) entropy loss. The loss functions have been inherited from DiffPool given that FPool is predicated on the DiffPool framework. Experimental testing and evaluation (including a comparative analysis, see Table 2 and Table 3) is set out in Section 5. However, there remain open research questions (ORQ) we have identified in our study:

1. *Is entropy loss necessary given that a GNN could handle a soft adjacency matrix which could be translated to a complete weighted graph*? The question, *could using additional entropy loss help improving performance?* requires further study.
2. As discussed in Section 5.2, GIN outperforms the alternative methods considered in the training time experiment (see Table 3). However, while GIN performs well in terms of the training time, it suffers from an inability to capture the hierarchical structure of many real-world datasets with lower accuracy (see Table 2). Addressing this dichotomy and improving the training time performance for *FPool* requires further investigation.
3. In Section 5.3, we introduced simulation results for FPool node clustering. However, there is a potential issue which relates to *how to learn the number of clusters to reduce parameters when both FPool [and DiffPool] complete the training successfully*? The issue (the potential for a significant number of redundant parameters) requires further research.

In future work, we will investigate the ORQ (points 1–3) as they relate to *FPool* with the aim of further improving (i) the classification performance for the *IMDB-Binary* dataset for which GIN is slightly better than FPool, and (ii) the computational overhead (the training time) which, as we have noted in Table 3, is slower than for FPool than is the case for GIN across all datasets.

## 7. Concluding Observations

This paper has considered the graph classification problem and the application of the GNN technique which is a popular and widely used DL approach for graph classification. In this paper, we propose FPool which is a novel method for graph classification based on the notion of hierarchical pooling to provide an effective method of capturing the hierarchical structure of graphs.

While FPool and DiffPool employ the same hierarchical pooling concept, our reported results show that FPool achieves improved classification performance in a comparative analysis. Moreover, we have evaluated the training time and FPool is significantly faster than DiffPool when dealing with sensor data sets. In our comparative analysis, all implementations have used the same sensor datasets and training methodology.

In conducting our study, we have identified a number of ORQ (see Section 6.1). Notwithstanding the ORQ, we posit that FPool provides an effective and efficient method for capturing the hierarchical structure of graphs with improved classification performance and training time.

## Figures and Tables

**Table 1:** The descriptive data for the benchmark datasets used in the experimental testing.

Benchmark Datasets				
Dataset	Number of Graphs	Number of Classes	Node Attribute Dimension	Contains Node Labels
Mutag [35]	188	2	0	Yes
Enzymes [31,36]	600	6	28	Yes
IMDB- Binary [37]	1000	2	0	No
D & D [38]	1178	2	0	Yes

**Table 2:** The relative percentage (%) classification accuracy for SAGPool, EdgePool, DiffPool, GIN, and FPool using the Mutag, Enzymes, IMDB-Binary, and D & D datasets.

Classification Accuracy Comparative Analysis				
	Mutag	Enzymes	IMDB-Binary	D & D
SAGPool	70.52 ± 2.58	21.00 ± 4.90	51.60 ± 5.61	69.49 ± 3.08
EdgePool	73.68 ± 4.71	36.67 ± 6.24	52.00 ± 5.48	72.03 ± 3.17
DiffPool	78.42 ± 10.90	44.00 ± 7.93	67.70 ± 5.29	74.84 ± 4.89
GIN	82.63 ± 10.00	54.83 ± 4.91	**68.20 ± 2.96**	70.09 ± 4.60
FPool	**84.21 ± 6.66**	**67.50 ± 7.97**	67.00 ± 2.45	**81.60 ± 0.48**

**Table 3:** The relative training time (in seconds per epoch) for SAGPool, EdgePool, DiffPool, GIN, and FPool using the Mutag, Enzymes, IMDB-Binary, and D & D datasets.

Training Time Comparative Analysis				
	Mutag	Enzymes	IMDB-Binary	D & D
SAGPool	0.166 ± 0.002	0.493 ± 0.005	0.804 ± 0.005	1.198 ± 0.011
EdgePool	0.280 ± 0.002	1.415 ± 0.019	2.472 ± 0.006	21.050 ± 0.336
DiffPool	0.296 ± 0.003	0.907 ± 0.043	1.365 ± 0.006	1.281 ± 0.095
GIN	**0.055 ± 0.003**	**0.136 ± 0.002**	**0.209 ± 0.001**	**0.270 ± 0.001**
FPool	0.171 ± 0.001	0.492 ± 0.003	0.731 ± 0.004	0.833 ± 0.059

## Data Availability

The study uses open and freely available data sources [datasets] identified and referenced in this paper. The data sources are reflected in other studies referenced in this study and in other papers published by MDPI (Sensors).
